# Supplementary material for: Optimal minimal residual disease threshold in pediatric acute myeloid leukemia: A retrospective cohort study based on the TARGET database
Source: PLoS Med. 2026 May 8;23(5):e1005088. doi: 10.1371/journal.pmed.1005088 (PMC13155632; doi:10.1371/journal.pmed.1005088)
Supplement: S1 Code — (ZIP) [file pmed.1005088.s002.zip › S2 code/PROJ8_16_tbl/PROJ8_16_tbl_0.pdf]

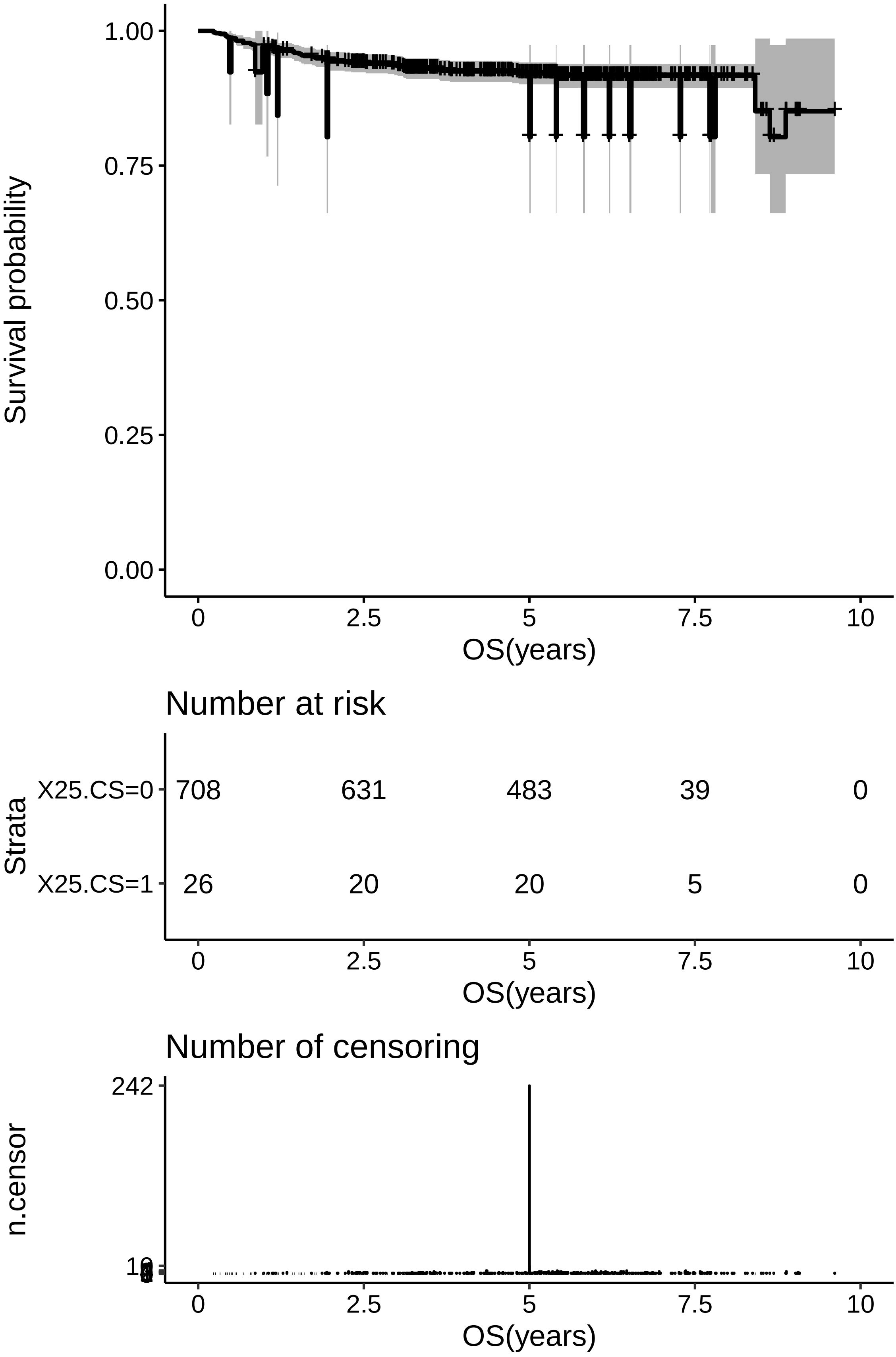

Survival probability
1.00
0.75
0.50
0.25
0.00
0
2.5
5
7.5
10
OS(years)
Number at risk
Strata
X25.CS=0 708 631 483 39 0
X25.CS=1 26 20 20 5 0
0
2.5
5
7.5
10
OS(years)
Number of censoring
n.censor
242
10
0
2.5
5
7.5
10
OS(years)
